# Phytochemical Analysis and Antioxidant, Antimicrobial, and Antibiofilm Effects of a New Himalayan Lichen *Placidium deosaiense* Usman and Khalid Growing in Pakistan

**DOI:** 10.3390/ijms252011203

**Published:** 2024-10-18

**Authors:** Anja Manojlović, Abdul Nasir Khalid, Muhammad Usman, Olgica Stefanović, Nevena Đukić, Nedeljko Manojlović, Jovica Tomović

**Affiliations:** 1 Department of Pharmacy, Faculty of Medical Sciences, University of Kragujevac, 34000 Kragujevac, Serbia; anjamanojlovic98@gmail.com (A.M.); mtnedeljko@gmail.com (N.M.); 2 Fungal Biology and Systematics Research Laboratory, Institute of Botany, University of the Punjab, Lahore 54590, Pakistan; nasir.botany@pu.edu.pk (A.N.K.); musmanmughal52@yahoo.com (M.U.); 3 School of Life Sciences, University of Nottingham, Nottingham NG7 2RD, UK; 4 Department of Biology and Ecology, Faculty of Science, University of Kragujevac, 34000 Kragujevac, Serbia; olgica.stefanovic@pmf.kg.ac.rs (O.S.); nevena.djukic@pmf.kg.ac.rs (N.Đ.); 5 Center for Harm Reduction of Biological and Chemical Hazards, Faculty of Medical Sciences, University of Kragujevac, 34000 Kragujevac, Serbia

**Keywords:** *Placidium deosaiense*, phytochemical analysis, antimicrobial, antibiofilm, antioxidant

## Abstract

Phytochemical composition and antimicrobial, antibiofilm, and antioxidant effects of a newly described Himalayan lichen *Placidium deosaiense* Usman and Khalid growing in Pakistan were investigated. HPLC–DAD methods were used for identification of secondary metabolites in acetone and methanol extracts. The total phenolics content was measured using a spectrophotometric method. The study investigated the antioxidant (DPPH-scavenging activity assay and reducing-power assay), antibacterial (minimum inhibitory concentration (MIC) and minimum bactericidal concentration (MBC)), and antibiofilm (inhibition of biofilm formation and reduction in mature biofilm) activities of extracts of the lichen *P. deosaiense* and isolated parietin. The chemical constituents olivetol, olivetolic acid, haematommic acid, fallacinol, and parietin were identified as major compounds in the tested extracts of the lichen. Parietin was isolated from the acetone extract on a separation column. The methanol extract had higher values of TPC (21.67 mg GAE/g) than the acetone extract. Isolated parietin showed the best antioxidant activity measures, according to the DPPH-scavenging activity assay (IC_50_ = 51.616 μg/mL) and reducing-power assay. Although the extracts showed the best antibacterial activity (especially against *Proteus mirabilis* ATCC 12453), parietin demonstrated superior antibiofilm activity (especially against *Staphylococcus aureus* ATCC 25923). This is the first report on the phytochemical composition of the lichen *Placidium deosaiense* and the first description of the chemical composition of some of the 45 species of the genus *Placidium*. This research will pave the way for further exploration of new activities of this lichen and its metabolites, which are important for medicine and pharmacy.

## 1. Introduction

A lichen represents a stable symbiotic relationship between a fungus and algae (typically green) and/or cyanobacteria. Lichens have the remarkable ability to produce over 1000 distinct secondary metabolites, which include monoaromatic compounds, anthraquinones, xanthones, dibenzofurans, depsones, depsides, and depsidones [1]. Many of these compounds are unique to lichens and exhibit various pharmaceutical activities. These include antioxidant, antiviral, antimicrobial, anti-inflammatory, and antiproliferative properties, as well as additional effects such as antipyretic, antiherbivore, allelopathic, and photoprotective activities [1,2,3,4,5]. *Placidium* is a small genus, and only 45 species have been reported worldwide [6,7,8,9]. *Placidium deosaiense* Usman and Khalid (*P. deosaiense*) was described in 2021 from the Deosai Plains and its adjacent areas, Gilgit Baltistan, Pakistan [8]. It grows on soil and falls into the group of Pyrenocarpous lichens of Ascomycota. After the Tibetan plateau, the Deosai Plains is the second highest alpine plateau in the world, and it comprises 2240 km^2^ of alpine tundra with an altitudinal range up to 5200 m above sea level. It is located in northern part of Pakistan between the Himalayas and Karakorum, i.e., the world-famous mountain ranges [10,11]. Annual precipitation ranges from 350 to 550 mm, with the majority falling as snow during winter [12]. The plateau’s ecosystem is marked by harsh cold, low atmospheric pressure, reduced oxygen and carbon dioxide levels, aridity, and intense, rapidly fluctuating ultraviolet solar radiation [13].

According to our knowledge, previous investigations of the chemistry of lichens from the genus *Placidium* have not confirmed the presence of any chemical compounds in them. Chemical analysis of the lichen *Placidium squamulosum* var. *argentinum* belonging to the Placidium genus showed that all tests for the presence of secondary metabolites have been negative [14]. Chemical analysis for the species *Placidium nitidulum*, *Placidium nigrum,* and *Placidium varium* found in China also showed that all the spot tests have been negative and that no substances have been detected by TLC [15].

The primary objective of this study was to assess, for the first time, the total phenolic content in the acetone and methanol extracts of *P. deosaiense* and to investigate their antioxidant, antimicrobial, and antibiofilm activities, highlighting their potential applications in medicine and pharmacy. Additionally, the phenolic compounds in *P. deosaiense* extracts were identified using high-performance liquid chromatography coupled with a photodiode array (HPLC–DAD).

## 2. Results

### 2.1. Phytochemical Analyses

**Figure 1:**
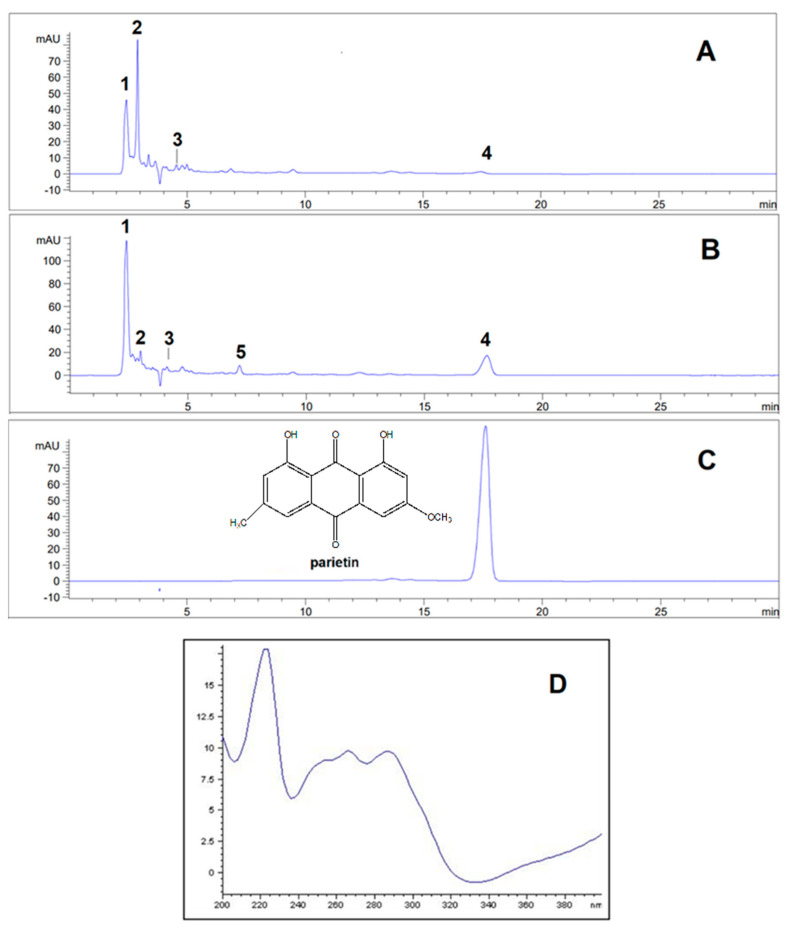
HPLC chromatograms of the acetone and methanol extracts of the lichen *P. deosaiense* and isolated parietin obtained at 254 nm as well as UV spectrum of parietin: 1—olivetol; 2—olivetolic acid (olivetil carboxylic acid); 3—haematommic acid. 4—parietin; 5—fallacinol. (**A**)—chromatogram of *Placidium deosaiense* acetone extract obtained at 254 nm. (**B**)—chromatogram of *Placidium deosaiense* methanol extract obtained at 254 nm. (**C**)—chromatogram of the isolated parietin. (**D**)—UV spectrum of parietin from 200 to 400 nm.

**Figure 2:**
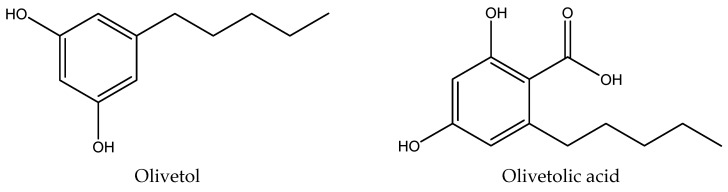

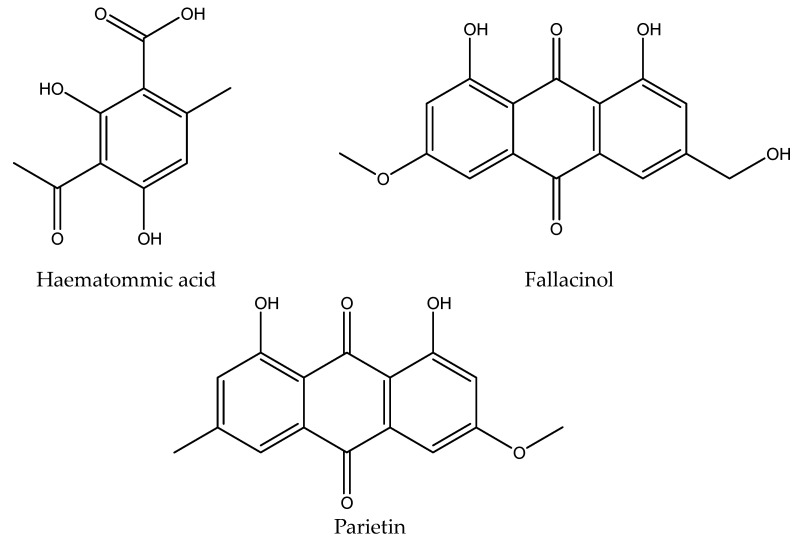
The chemical structures of compounds identified in extracts.

HPLC chromatograms of the acetone and methanol extracts of *P. deosaiense*, a lichen collected in the Himalayas in Pakistan, as well as secondary metabolite parietin, are presented in Figure 1. A total of five different compounds (olivetol, olivetolic acid, haematommic acid, fallacinol, and parietin) were identified in extracts, with olivetol being the most abundant compound (Table 1). The relative abundance of the identified compounds between the extracts was statistically significantly different (*p* < 0.05). Structures of all of the detected compounds are shown in Figure 2. In addition, parietin was isolated from the acetone extract on a separation column using different separation systems and used for further tests.

### 2.2. Yield of the Extraction, Total Phenolic Content, and the Amount of Parietin

The data presented in Table 2 indicate that higher yields were achieved by extracting *P. deosaiense* lichen with the methanol compared to the acetone (statistically significant differences (*p* < 0.05)). Quantitative analysis performed using the HPLC method showed that the amount of parietin in the methanol extract was 0.310 mg/mL, while the amount of this compound in the acetone extract was about ten times less and amounted to 0.028 mg/mL (significant differences (*p* < 0.05)).

Table 2 shows the results of the total phenolic content (TPC) in the acetone and methanol extracts of the tested lichen. The methanol extract demonstrated statistically significantly higher TPC (21.67 mg GAE/g) values compared to the acetone extract (*p* < 0.05).

### 2.3. Antioxidant Activity

The antioxidant activity of two *P. deosaiense* extracts and the isolated compound parietin was assessed using two methods: DPPH-free-radical-scavenging activity assay and reducing-power assay (Table 3). The evaluation of the antioxidant activity revealed that all tested extracts were effective in scavenging the DPPH radical. The isolated compound parietin showed significantly higher DPPH-free-radical-scavenging activity (IC_50_ = 51.616 µg/mL) than the tested extracts of the lichen *P. deosaiense* (*p* < 0.05). The acetone and methanol extracts of the lichen *P. deosaiense* demonstrated comparable efficacy in the scavenging of DPPH radicals (no statistically significant differences among the extracts). The results of the reducing power for the tested lichen extracts and parietin are presented in Table 3. The data show that the extracts and parietin exhibited lower activity compared to ascorbic acid (statistically significant differences among the extracts, parietin and ascorbic acid (*p* < 0.05)), at higher concentrations, but at lower concentrations, the difference in the activity was not so pronounced (no statistically significant differences among the extracts, parietin and positive control). Parietin exhibits the highest reducing power, while the acetone extract displays the lowest reducing power at higher concentrations. However, at lower concentrations, the difference between the acetone extract and the other tested extracts, including parietin, is smaller and not statistically significant.

### 2.4. Antibacterial Activity

Table 4 displays the in vitro antibacterial activity results for the acetone extract, methanol extract, parietin, and the positive control (tetracycline). In this experiment, the minimum inhibitory concentration (MIC) and the minimum bactericidal concentration (MBC) values ranged from 1.25 to >10 mg/mL for the acetone extract, methanol extract, and parietin, and from <0.25 to >128 µg/mL for tetracycline. Statistically significant differences in activity among the extracts and parietin were not observed (*p* > 0.05). The results of testing the antimicrobial activity of the acetone and methanol extracts of *P. deosaiense* lichen and parietin showed that the best antibacterial activity was recorded in relation to *Proteus mirabilis* ATCC 12453 and *Bacillus cereus* ATCC 11778. The analyzed extracts of the lichen *P. deosaiense* and parietin showed weak antibacterial activity on the other tested bacterial species. Based on the findings, it can be inferred that the methanol extract of *P. deosaiense* exhibited superior antibacterial activity (MIC = 1.25 mg/mL) against *Proteus mirabilis* ATCC 12453 compared to the acetone extract (MIC = 2.5 mg/mL) and parietin (MIC = >10 mg/mL). The acetone extract showed better antibacterial activity (MIC = 5 mg/mL; MBC = 5 mg/mL) against *Bacillus cereus* ATCC 11778 than the methanol extract (MIC = 10 mg/mL; MBC = 10 mg/mL) and parietin (MIC and MBC = >10 mg/mL). Parietin exhibited minimal antibacterial activity against all tested bacterial species (MIC and MBC = >10 mg/mL). When compared to the positive control (tetracycline), the tested extracts and parietin exhibited limited (parietin) to moderate (the methanol and acetone extracts) effects against certain bacterial species. Statistically significant differences in activity among the extracts and antibiotic (*p* = 0.002) and between parietin and antibiotic (*p* = 0.0001) were observed.

### 2.5. Antibiofilm Activity

To evaluate the in vitro antibiofilm activity of the acetone and methanol extracts of *P. deosainse* and parietin, inhibition of biofilm formation and reduction in mature biofilm was investigated. The antibiofilm effect of the extracts and parietin on the *P. mirabilis* ATCC 12453, *P. aeruginosa* ATCC 10145, and *S. aureus* ATCC 25923 biofilm formation were examined. The results (Table 5 and Table 6) show the influence of different concentrations on the percentage of biofilm inhibition and reduction. The solvent control (5% DMSO) did not affect bacterial growth. Statistically significant differences in activity among the extracts and parietin were not observed (*p* > 0.05). The examined extracts of *P. deosainse* and parietin showed the greatest inhibition of biofilm formation *S. aureus* ATCC 25923. Parietin exhibited the best inhibition of biofilm formation *S. aureus* ATCC 25923, mostly at all concentrations (range: from 99.6% to 63.7%). Parietin demonstrates strong inhibition of biofilm formation of *P. mirabilis* ATCC 12453 (10 mg/mL—92.5%) and *P. aeruginosa* ATCC 10145 (10 mg/mL—99.8%), but only at higher concentrations. The acetone extract was more effective than the methanol extract. At the highest concentration (10 mg/mL), the acetone extract inhibited 92.1% of *P. aeruginosa* ATCC 10145 and 92% of *S. aureus* ATCC 25923. The acetone extract showed a strong inhibition of the formation of *S. aureus* ATCC 25923 biofilm and at lower concentrations (1.25 mg/mL, 2.5 mg/mL, and 5 mg/mL) over 90%. The methanol extract also inhibited the biofilm formation of *S. aureus* ATCC 25923 at all tested concentrations. At concentrations of 5 mg/mL and 10 mg/mL, the methanol extract also inhibited the biofilm of *P. aeruginosa* ATCC 10145, and at the concentration of 10 mg/mL, it had an inhibitory effect on the formation of *P. mirabilis* ATCC 12453 biofilm.

Analysis of the effect of the acetone and methanol extracts of *P. deosainse* and parietin on the reduction in mature biofilms (Table 6) was more moderate. The maximum reduction was achieved in relation to the mature biofilm *S. aureus* ATCC 25923 (up to 85.3% reduction by parietin; 75.5% reduction by the methanol extract; and 69.8% reduction by the acetone extract). The reduction in the mature biofilm *P. mirabilis* ATCC 12453 is up to 20.4% by the methanol extract, but the same extract had no effect on inhibition of mature biofilm of *P. aeruginosa* ATCC 10145. The mature biofilm of *P. aeruginosa* ATCC 10145 was inhibited by the acetone extract up to 28.2% and parietin up to 59.8%.

## 3. Discussion

The present paper, to the best of the authors’ knowledge, is the first to deal with the chemical analyses of the acetone and methanol extracts of a novel arctic alpine lichen, *Placidium deosaiense,* as well as the investigation of in vitro antimicrobial, antibiofilm, and antioxidant effects of the above-mentioned extracts and isolated parietin.

As can be seen in the HPLC chromatogram of the acetone extract (Figure 1), five different compounds were identified for the first time. The signal at retention time 2.42 min originated from olivetol. Olivetol belongs to the monoaromatic compound, as well as olivetolic acid whose signal is located at 2.91 min. Olivetolic acid (olivetol carboxylic acid) is a carboxylated derivative of olivetol, and these two compounds do not occur so often in lichens and are not widely distributed. These two compounds have been reported from lichens *Cetrelia monachorum* and *Ramalina conduplicans* and their biological activity, including anti-inflammatory, antioxidant (DPPH and ABTS scavenging, protection against hydroxyl radical-induced DNA damage), antihyperglycemic, antimicrobial, antitumor, antiviral activities, and others, has been extensively investigated [16,17,18,19]. Olivetol is a compound known to occur in some lichen species [16] but is best known as a precursor of tetrahydrocannabinol, found in *Cannabis sativa* [20]. The small signal that occurs at 4.56 min comes from haematommic acid, which is a common metabolite in lichens [21]. The signal with weak-medium intensity comes from the anthraquinone parietin, and it appears at 17.54 min. Its structure was confirmed on the basis of the retention time values and the UV spectrum of a standard substance previously isolated from the lichen *Xanthoria parietina* [22]. It is mostly characteristic of lichen genera *Xanthoria*, *Teloschistes*, and *Caloplaca* [23], and this is the first time it has been found in the genus *Placidum*. In contrast to the chromatogram of the acetone extract, the signal originating from parietin was more intense. Given the photoprotective and protective role of parietin [24], which is very important for the survival of this lichen under extreme external influences [25], its amount in the extracts was determined. It is an orange anthraquinone pigment, which is characteristic for sun-exposed habitats. This is the most likely explanation for its unexpected presence in this lichen that grows at 5200 m above sea level and is exposed to a large amount of sunlight.

Methanol often provides a higher yield of extraction compared to acetone in lichens due to several factors related to its chemical properties and the nature of the compounds present in lichens. Many bioactive compounds in lichens, such as phenolics, are polar and dissolve better in polar solvents like methanol, which enhances the extraction yield of these compounds [26]. Parietin is a polar compound, and methanol, being a highly polar solvent, is more effective in dissolving and extracting polar compounds. This higher polarity of the methanol allows it to solubilize parietin more efficiently than the acetone. Methanol has a smaller molecular size and better penetration capabilities, which allows it to break down the cell walls of lichens more effectively. This results in a higher release of intracellular compounds, including parietin [27].

While several studies have investigated the antioxidant potential of various lichen species [28,29], this is the first study to explore the antioxidant activity of the novel lichen *P. deosaiense*. Other authors have examined lichen extracts that contained metabolites that were also identified in our samples. Taslimi and Gulçin evaluated the antioxidant properties of olivetol using various methods. The IC_50_ values of olivetol in the DPPH^•^, ABTS^•+^, DMPD^•+^,O_2_^•−^, and metal-chelating assays were 17.77, 1.94, 19.25, 53.30, and 2.83 μg/mL, respectively [30]. The tested extracts of *P. deosaiense* lichen as well as isolated parietin showed the ability to scavenge DPPH radicals as well as reducing power, whereby isolated parietin showed the best antioxidant activity. The examination of parietin as an antioxidant agent has already been examined in other studies, as well as the other metabolites present in the examined extracts [31,32]. In tests by other authors, the antioxidant activity mostly depended on the total phenols present and the lichen components themselves (depside, depsidone, tridepside, and other phenolic components). In most studies, total phenolic content was positively correlated with antioxidant activity [33,34].

In vitro evaluations of various lichens against human pathogenic bacteria have been conducted [35]. Recent research has shown that the methanol extract of *Placidium squamulosum* showed high antibacterial activity against all seven tested bacterial strains (*E. coli* ATCC1652, *S. typhi* ATCC1679, *P. mirabilis* ATCC2601, *S. aureus* ATCC1885, *E. faecalis* ATCC2321, *S. epidermidis* ATCC2405, and *B. cereus* ATCC13061), with an MIC value from of 250 to 500 mg/mL [36]. However, our study is the first to investigate the antimicrobial activity of *P. deosaiense* extracts. The best antibacterial activity of the tested extracts in our work was shown for the species *P. mirabilis* ATCC 12453 and *B. cereus* ATCC 11778, and much weaker results were observed for the other tested bacterial lines. The relative resistance of *E. coli*, *S. aureus*, *P. aeruginosa*, *E. faecalis* compared to *P. mirabilis* and *B. cereus* can be attributed to several factors related to their intrinsic and acquired resistance mechanisms: genetic adaptability, biofilm formation, efflux pumps, enzymatic degradation, and cell wall structure [37]. The acetone and methanol extracts of *P. deosaiense* demonstrated superior antibacterial activity compared to isolated parietin. This finding aligns with other studies, indicating that acetone and methanol extracts of lichens generally exhibit better antimicrobial activity than isolated compounds. This enhanced activity is attributed to the complex mixture of bioactive compounds in the extracts, which can work synergistically to produce a more potent antimicrobial effect. Methanol, being a polar solvent, is particularly effective at extracting phenolic compounds and flavonoids, which are known for their antimicrobial properties. This complex mixture in extracts can target multiple microbial pathways, enhancing overall antimicrobial efficacy compared to a single isolated compound [38,39,40]. Isolated parietin showed weak antibacterial activity against the tested bacterial lines. Basile et al. examined the antimicrobial activity of acetone extract of *Xanthoria parietina* and parietin. The extract and parietin were assessed for antimicrobial activity against nine standard bacterial strains from the American Type Culture Collection (ATCC) and various clinically isolated strains. Both samples demonstrated significant antibacterial activity across all tested strains and clinical isolates, showing particularly strong effectiveness against *S. aureus* from both standard and clinical sources [41]. Also, parietin and fallacinol, isolated from *Caloplaca lactea* extract, showed potent antibacterial activity [42]. In relation to our testing of the antibacterial activity, they were performed on different bacterial lines and ATCC strains. A recent study has revealed a mode of antibacterial action of anthraquinones. Anthraquinones exert their antibacterial activity by inhibiting proteins or nucleic acids synthesis, disturbing the integrity of the bacterial cell membrane and cell wall, inhibiting the respiratory metabolic process or regulating efflux pumps, enzymes, and active oxygen species activity [43].

To the best of the authors’ knowledge, the antibiofilm activity of *P. deosaiense* extracts has not been investigated previously, although there is an existing study on the antibiofilm activity of parietin. In that study, the antibiofilm activity of parietin has been demonstrated against *S. aureus* and *E. faecalis* [44], which is particularly significant since biofilms are involved in 80% of human microbial infections. Anthraquinone derivatives, frequently occurring in plants, are noted for their antimicrobial activities through multiple mechanisms. However, they are also associated with toxic and laxative effects, which could pose challenges and result in undesirable side effects in pharmaceutical applications [45]. In a study by Mitrovic et al., antibiofilm potentials (against *S. aureus* ATCC 25923 and *P. mirabilis* ATCC 12453) of extracts from the lichen species *Platismatia glauca* and *Pseudevernia furfuracea* were evaluated. GC–MS analyses of the extracts led to the identification of olivetol (which is also in high abundance in our examined extracts). The acetone extracts of *Platismatia glauca* and *Pseudevernia furfuracea* showed better antibiofilm activity than their methanol extract (as well as our examined extracts, but they had a weaker ability of inhibition) [46].

Biofilm is an aggregation of bacteria within extracellular polymer matrices that adhere to either biotic or abiotic surfaces, conferring greater resistance to varying environmental conditions and biocides compared to the planktonic form of bacteria [47].

In the present study, the acetone and methanol extracts and parietin from *P. deosaiense* exhibit dual actions by both preventing biofilm formation and eradicating mature biofilms. Although the extracts showed the best antibacterial activity, parietin demonstrated a superior antibiofilm effect. This suggests that lichen metabolites are the primary contributors to the antibiofilm properties of the tested extracts. It should be noted that the tested extracts and parietin exhibited better antibiofilm effects against the Gram-positive strain *S. aureus* at all concentrations, while their effects were weaker against the Gram-negative strains *P. aeruginosa* and *P. mirabilis*. The exception was at higher concentrations, where there was significant biofilm inhibition against *P. aeruginosa*. The antibiofilm activity of the tested extracts and parietin is particularly effective against Gram-positive bacteria such as *S. aureus*, likely due to their cell wall structure. Gram-positive bacteria have a thick peptidoglycan layer, which is a primary target for many antimicrobial agents found in lichen extracts. These extracts can effectively disrupt the synthesis and integrity of the peptidoglycan layer, impairing the bacteria’s ability to form and maintain biofilms [39,40]. The biofilm eradication ability of *P. deosaiense* extracts and parietin was reduced but remained significant against *S. aureus* biofilm. The literature does not offer a precise explanation for the mechanism behind the antibiofilm activity of lichens and their extracts. However, it is generally believed that secondary metabolites of lichens are responsible for both their antibiofilm and antimicrobial effects [46,48]. Several lichen compounds, including usnic acid, atranorin, evernic acid, psoromic acid, and butyrolactone analogs, have demonstrated antibiofilm activity [48,49,50].

## 4. Materials and Methods

### 4.1. Materials with Harvested Lichen

**Figure 3:**
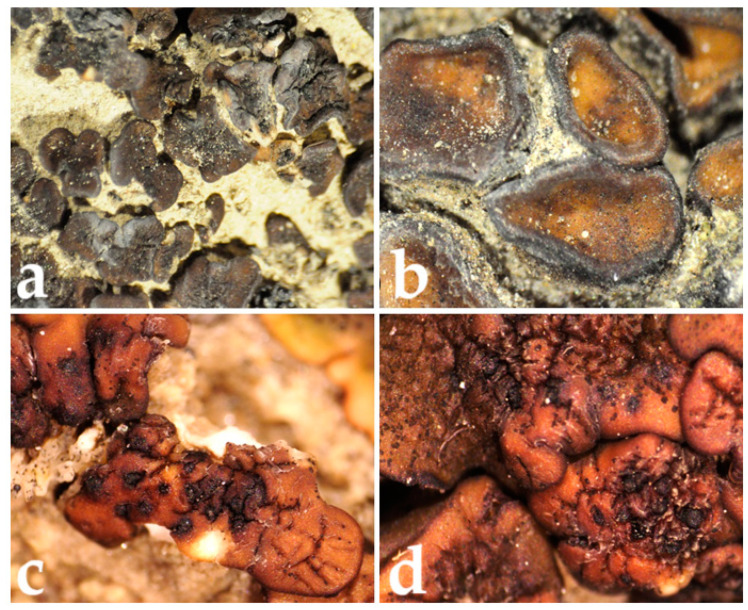
Lichen *Placidium deosaiense* Usman and Khalid sp. nov. holotype (LAH36819). (**a**,**b**): Dry form. (**c**,**d**): Wet form.

Lichen was collected from Deosai National Park in the Gilgit–Baltistan region of the Islamic Republic of Pakistan (35°01′32.5″ N; 75°22′22.5″ E; 4057 m; on soil) (Figure 3). Specimens of the types of lichen *Placidium deosaiense* Usman and Khalid were determined at the Institute of Botany, University of the Punjab, Lahore (LAH Herbarium; voucher number: LAH36819) using the relevant key and monographs. *P. deosaiense* is characterized by 2–7 mm wide squamules, brown to blackish upper surface, epinecral layer up to 70 µm thick, pale exciple except for the ostiolum, larger cylindrical asci 84.9–108.5 × 6.9–12.2 µm, and clavate to bacilliform pycnidiospores 4.4 ± 0.4 × 1.8 ± 0.3 µm [8,10,11,51].

The lichen material was cleaned from impurities (soil, pebbles, other lichens, etc.), dried at 20–25 °C, and then ground (pulverized). Twenty grams of the lichen were ground, and half of this amount (10 g each) was placed in two 250 mL Erlenmeyer flasks. In total, 150 mL of the acetone and methanol were poured into each Erlenmeyer flask. The Erlenmeyer flasks were closed and left to stand for 48 h at room temperature with constant stirring. After that, both contents were filtered. The liquid part was evaporated on a rotary vacuum evaporator until dry. The dry extracts were weighed, and the percentage yield of extract was calculated gravimetrically using the dry weight of concentrate (a) and the plant dry weight (b) as follows: %yield = a/b.

Acetone, methanol, and toluene used for extraction and isolation were of analytical grade and were purchased from Fisher, Leicestershire, UK. For HPLC, experimental water was generated using a Milli-Q water purification system (Milford, MA, USA), methanol was HPLC grade (Merck, Darmstadt, Germany), and phosphoric acid was the analytical reagent grade (Sigma Aldrich GmbH, Sternheim, Germany). 1,1-Diphenyl-2-picrylhydrazylhydrate (DPPH), Folin–Ciocalteu, ascorbic acid, gallic acid, and tetracycline were purchased from Sigma (Sigma-Aldrich GmbH, Sternheim, Germany). Potassium ferricyanide, phosphate buffer, FeCl_3_, and resazurin were purchased from Alfa Aesar (Alfa Aesar GmbH & Co., Karlsruhe, Germany). Gram-negative bacteria *Escherichia coli* ATCC 25922, *Proteus mirabilis* ATCC 12453, *Pseudomonas aeruginosa* ATCC 10145, Gram-positive bacteria *Staphylococcus aureus* ATCC 25923, methicillin-resistant *Staphylococcus aureus* ATCC 43300, *Enterococcus faecalis* ATCC 29212, and *Bacillus cereus* ATCC 11778 were used.

### 4.2. Phytochemical Analyses

#### 4.2.1. HPLC (High-Performance Liquid Chromatography)

HPLC was utilized to analyze and identify the individual components of the extracts. Analyzes were conducted using the Agilent 1200 Series (Agilent Technologies, Santa Clara, CA, USA) using the C18 column (ZORBAX Eclipse XDB-C18; 25 cm × 4.6 mm; 5 μm). Separate detection of dots was carried out using a Diode Array Detector (DAD) at wavelengths of 280, 330, and 350 nm, while the absorption spectra of the components were recorded over a range of 200 to 400 nm. Dissolved solubilized samples were filtered through using a pore size of 0.45 μm. Chromatographic separation was performed using a solvent system of methanol, water, and phosphoric acid in the ratio of 85:15:0.9 (*v*/*v*/*v*). The mobile phase flow rate was 1 mL/min, and the injected sample amount was 10 μL. The column was thermostated at a temperature of 30 °C. This procedure was previously explained and used [52,53]. Chromatograms and UV spectral data were obtained at a wavelength of 254 nm. The identification of the secondary metabolites of the acetone and methanol extracts of the lichen *P. deosaiense* was accomplished by comparing the retention times (t*_R_*) and UV spectra of the metabolites with those of standard references (λ = 200–400 nm). The standards employed for HPLC identification were obtained from the following sources: olivetol and olivetolic acid from the lichen Cladonia macaronesica, haematommic acid from Umbilicaria crustulosa, and parietin and fallacinol from the lichen Xanthoria parietina. The standard compounds have been previously isolated in our laboratory and their structures were confirmed by mass spectrometry, ^1^H and ^13^C-NMR.

#### 4.2.2. Isolation of Parietin

A measured quantity of 10 g of the lichen was immersed in 150 mL of acetone and macerated for 48 h with constant stirring. After that, the entire amount was filtered through filter paper, and the liquid part evaporated on a vacuum evaporator under reduced pressure. The extract was dissolved with a small amount of eluent and silica gel, and then it was applied at the top to the column and separated by column chromatography on Silica Gel 60 (0.063–0.200 mm; column length 25 cm; internal diameter 3 cm) using the following solvent systems: toluene, toluene–acetone (80:20, 60:40, 40:60, *v*/*v*), and acetone, respectively. The first eluted compound from the column was parietin. After collection of fractions in an Erlenmeyer flask and evaporation, the residue was recrystallized in the acetone to give orange crystals. Its structure was confirmed on the basis of spectral values by comparison with literature data [54]. The purity of the isolated parietin was determined by HPLC–DAD and amounted to 98.7%. Parietin was further used for the antioxidant, antimicrobial, and antibiofilm testing.

#### 4.2.3. Determination of Total Phenolic Content (TPC)

To determine the content of total phenolic compounds in tested extracts the method described by Singleton et al. was employed [55]. Briefly, 1 mL of the extracts at a concentration of 0.25 mL was mixed with 5 mL of 10-times diluted Folin–Ciocalteu reagent and 4 mL of 7.5% NaHCO_3_. After 15 min of incubation at room temperature, the absorbance of the mixtures was read at 765 nm using UV–Vis double beam spectrophotometer Halo DB-20S (Dynamica GmbH, Dietikon, Switzerland). All measurements were conducted in triplicate. The TPC values were expressed as mg equivalents of gallic acid per 1 g dry extract (mg GAE/g).

### 4.3. Antioxidant Activity

#### 4.3.1. DPPH-Free-Radical-Scavenging Activity Assay

To determine the ability to neutralize DPPH radicals, we used the method described by Takao et al. [56]. The method is based on the principle of a hydrogen donor as an antioxidant, whereby the ability to neutralize free radicals is measured. In the neutralization reaction, after taking over a hydrogen atom from the antioxidant, the stable compound 2,2-diph-enyl-1-picrylhydrazyl is obtained, and the color changes from purple to yellow. Antioxidant activity is proportional to the decrease in absorbance measured spectrophotometrically at 517 nm. A series of 10 double dilutions with a volume of 2 mL was made from the sample solutions (extracts and standards). In total, 2 mL of 40 mM DPPH solution was added to 2 mL of the sample. In comparison, a control was prepared with methanol instead of the sample. Then, the reaction mixture was left in the dark for 30 min, and the ability to neutralize DPPH radicals was determined by measuring the absorbance at 517 nm. All working trials and controls were tested in triplicate. Ascorbic acid was used as the positive controls. The scavenging capacity of DPPH free radical was calculated according to the following Equation (1):

Scavenging capacity of DPPH free radical (%) = Ac − As/Ac × 100,(1)

The Ac-absorbance of the control solution (negative control): As is the absorbance of the sample solution or standard. The dependence curve of the percentage of neutralization on the concentration of the samples was used to calculate the IC_50_ value, which is defined as the concentration of the tested sample that inhibits the action of free radicals by 50%.

#### 4.3.2. Reducing-Power Assay

To determine the reductive activity (reducing capacity or power) of the lichen extracts, we used the ferricyanide/Prussian blue method described by Oyaizu [57]. The method is based on the reduction of Fe^3+^ to Fe^2+^ in the presence of antioxidants in acidic conditions, whereby the complex known as Prussian blue with λmax = 700 nm is formed. A series of 10 double dilutions with a volume of 1 mL was made from the sample solution (extract and standard). At the same time, a control with methanol was prepared instead of the sample. In total, 2.5 mL of phosphate buffer (0.2 M, pH 6.6) and 2.5 mL of 1% potassium ferricyanide were added to all samples (extract, standard, control). The mixture was incubated for 20 min at a temperature of 50 °C. Then, 2.5 mL of 10% TCA was added to the mixture. Centrifugation followed. After centrifugation, two layers separated. In total, 2.5 mL of the supernatant was taken and added to 2.5 mL of distilled water and 0.5 mL of FeCl_3_. The absorbance of the solution was measured at 700 nm on a spectrophotometer. As a standard (positive control), ascorbic acid was used to compare the activity. The increase in the absorbance of the mixture solution shows how much the reducing power is increased. All measurements were repeated three times, and the results are shown as mean value ± standard deviation. A higher absorbance value indicates a higher reductive capacity of the sample.

### 4.4. Antibacterial Activity

#### 4.4.1. Samples and Controls

Stock solutions of the extracts and parietin at a concentration of 20 mg/mL were prepared in DMSO and then diluted in Mueller–Hinton broth (Torlak, Belgrade, Serbia) to achieve a 10% DMSO. As a positive control, antibiotic tetracycline dissolved in Mueller–Hinton broth was used. A negative control solvent was used (5% DMSO and lower). Each experiment included growth control (broth + bacterium) and sterility control (broth + extract).

#### 4.4.2. Bacterial Inoculum Preparation

Bacterial inocula were prepared by a direct colony suspension method and adjusted to 0.5 McFarland turbidity standard using a densitometer (DEN-1, BIOSAN, Riga, Latvia) [58].

#### 4.4.3. Determination of Minimum Inhibitory Concentrations (MICs)

MICs were determined according to the broth microdilution method established by the Clinical and Laboratory Standards Institute (CLSI, 2012) [59]. Two-fold serially diluted stock concentrations of tested extracts and parietin were prepared with Mueller–Hinton broth in the concentration range of 10 to 0.3125 mg/mL. A total of 10 µL of diluted bacterial suspension to achieve a final inoculum size of 5 × 10^6^ colony-forming units (CFUs)/mL were added to 100 µL of samples. The prepared 96-well microtiter plates were incubated at 37 °C for 20 h and then re-incubated for 2 h after adding 5 µL of resazurin solution, the indicator of microbial growth. MIC values were defined as the lowest concentration of tested extracts that prevented resazurin color change from blue to pink.

#### 4.4.4. Determination of Minimum Bactericidal Concentrations (MBCs)

After testing the growth assay to assess the MICs, the MBC test was performed by subculturing 10 µL aliquots from wells with no color change on nutrient agar plates (Torlak, Belgrade, Serbia). The lowest extract concentration at which no bacterial growth was observed after overnight incubation was considered MBC. MBC values were determined by subculturing 10 µL aliquots from wells with no color change on nutrient agar plates (Torlak, Belgrade, Serbia). The lowest extract concentration at which no bacterial growth was observed after overnight incubation was considered MBC.

### 4.5. Determination of Antibiofilm Activity

#### 4.5.1. Samples and Controls

Stock solutions of the extracts and parietin at a concentration of 20 mg/mL were prepared in DMSO and then diluted in Mueller–Hinton broth (Torlak, Belgrade, Serbia) to achieve a 10% DMSO. A negative control solvent was used (5% DMSO and lower). Each experiment included growth control (broth + bacterium), extract control (broth + extract), and broth control (broth only).

#### 4.5.2. Bacterial Inoculum Preparation

Three biofilm-positive bacterial strains, Proteus mirabilis ATCC 12453, Pseudomonas aeruginosa ATCC 10145, and Staphylococcus aureus ATCC 25923, were chosen. Their ability of in vitro biofilm formation was confirmed according to Stepanović et al. [58]. Bacterial inocula were prepared by a direct colony suspension method and adjusted to 0.5 McFarland turbidity standard using a densitometer.

#### 4.5.3. Inhibition of Biofilm Formation

Following a crystal violet assay, the effect of extracts and parietin on biofilm formation was evaluated in 96-well flat-bottomed polystyrene tissue culture (TC)-treated microtiter plates. Tryptic soy broth (TSB) (Torlak, Belgrade, Serbia), supplemented with additional glucose to a final concentration of 1%, was used as a nutrient broth. Two-fold serial dilutions of the extracts and parietin were prepared resulting in a decreasing concentration range (10–0.3125 mg/mL). Optical densities (ODs) of the samples were measured at 550 nm using a microplate reader (RT-2100C, Rayto, Shenzhen, China). The percentage of biofilm inhibition was calculated using the following formula presented in Ali et al. [60]:

(2) $$\mathrm{Inhibition}\;(\%)=\frac{\left({\mathrm{OD}}_{\mathrm{GC}}-{\mathrm{OD}}_{B}\right)-\left({\mathrm{OD}}_{S}-{\mathrm{OD}}_{\mathrm{EC}}\right)}{\left({\mathrm{OD}}_{\mathrm{GC}}-{\mathrm{OD}}_{B}\right)}\times 100$$

where OD_GC_ is the OD value of the growth control, OD_B_ is the OD value of the broth control, OD_S_ is the OD value of the sample, and OD_EC_ is the OD value of the extract control.

#### 4.5.4. Inhibition of Formed Biofilm

In order to examine the potential effect of the extracts and parietin on formed biofilm, 20 μL of bacterial suspension of each strain was first inoculated in 180 μL of TSB with 1% glucose in 96-well flat-bottomed TC-treated microtiter plates and incubated at 37 °C for 20 h without adding extracts. After incubation, free-floating bacteria were removed, and the wells were rinsed. Then, formed biofilms were treated with 100 μL of the extracts and parietin at varying concentrations (10–0.3125 mg/mL) and incubated further for 24 h. The biofilms were stained according to the method previously described. The OD values of samples were measured at 550 nm using an ELISA plate reader. The percentage of reduction in biofilm biomass was calculated using the Formula (2).

### 4.6. Statistical Analysis

The data are expressed as the mean ± standard deviation (SD) from three different measurements where it was appropriated. ANOVA analysis was used to compare the different groups. The results were considered statistically significant if *p* < 0.05. Microsoft Excel (Microsoft Excel^®^ version 2013, Microsoft Co., Ltd., Redmond, WA, USA) was used for generating graphs and calibration curves. Commercial IBM SPSS version 20.0 for Windows was used for all other statistical analysis.

## 5. Conclusions

According to our knowledge, previous scientific research has not dealt with the chemical analysis of species from the genus *Placidium*. Five secondary metabolites, olivetol, olivetolic acid, haematommic acid, fallacinol, and parietin as major compounds were identified in *Placidium deosaiense* Usman and Khalid using the HPLC–DAD method. The special chemotaxonomic importance of this work lies in the fact that for the first time the chemical composition of some *Placidium* species was analyzed.

This study also showed that the tested lichen has important quantities of phenolic compounds. Parietin was isolated from the acetone extract on a separation column. It was of particular importance to show the antimicrobial, antibiofilm, and antioxidant activities of this specific and new species, given that it grows under specific conditions at high altitude (5200 m above sea level). Testing the activity of the acetone and methanol extract of the lichen *P. deosaiense* showed its antibacterial and antioxidant properties, which were tested for the first time. The isolated compound parietin demonstrated the strongest DPPH free-radical-scavenging activity (IC_50_ = 51.616 µg/mL) and the highest reducing capacity compared to the tested extracts of the lichen *P. deosaiense*. Although the extracts showed the best antibacterial activity (especially against *Proteus mirabilis* ATCC 12453), parietin demonstrated superior antibiofilm activity (especially against *Staphylococcus aureus* ATCC 25923). Based on the obtained results, it can be assumed that the specific secondary metabolites identified in the new species probably play an important protective role against the extreme factors of the external environment in which this lichen grows. This research will serve for further examination of new activities of this lichen and its metabolites of importance for medicine and pharmacy.

## Figures and Tables

**Table 1:** Retention time, absorbance maxima, and relative abundance of the examined lichen substances.

Sr. No	Compound	Retention Time (t_R_ ± SD) * (min)	Absorbance Maxima (nm)	Relative Abundance % (254 nm)	
				Extracts	
				Acetone ^a^	Methanol ^b^
1	Olivetol	2.42 ± 0.01	278	20.3995	34.0514
2	Olivetolic acid	2.91 ± 0.02	218, 263, 301	25.8901	4.0678
3	Haematommic acid	4.56 ± 0.01	202, 237, 259	5.3064	2.2663
4	Parietin	17.54 ± 0.02	222, 266, 286, 438	1.0588	10.7476
5	Fallacinol	7.20 ± 0.01	223, 325, 435	**/	1.6712

* Values are the means of three determinations ± SD; **/—not detected; different superscript letters mean statistically significant differences among the extracts (*p* < 0.05).

**Table 2:** The yield of the extraction, total polyphenol content of the extracts of the lichen *P. deosaiense,* and the amount of parietin in the extracts.

Lichen Extracts	Yield (%)	Total Phenolics Content (mg GAE/g) *	Amount of Parietin (mg/mL)
Acetone Methanol	0.676 ^a^	19.46 ± 0.75 ^a^	0.028 ^a^
	1.063 ^b^	21.67 ± 0.41 ^b^	0.310 ^b^

* Values are expressed as mean ± SD of triplicate measurements; GAE—gallic acid equivalents; means with different letters in the same column indicate statistically significant (*p* < 0.05) differences among the extracts.

**Table 3:** The antioxidant activity (DPPH-scavenging assay and reducing-power assay) of the extracts of the lichen *P. deosaiense* and parietin.

Lichen Extract/ Compound	DPPH-Scavenging Assay IC_50_ (μg/mL)	Reducing-Power Assay Absorbance (700 nm)					
		1000 μg/mL	500 μg/mL	250 μg/mL	125 μg/mL	62.5 μg/mL	31.25 μg/mL
Acetone	270.22 ± 4.80 ^a^	0.074 ± 0.001 ^a^	0.059 ± 0.001 ^a^	0.060 ± 0.001 ^a^	0.056 ± 0.001 ^a^	0.055 ± 0.001 ^a^	0.053 ± 0.002 ^a^
Methanol	275.124 ± 9.713 ^a^	0.0853 ± 0.001 ^a^	0.066 ± 0.004 ^a^	0.066 ± 0.001 ^a^	0.062 ± 0.002 ^a^	0.062 ± 0.002 ^a^	0.061 ± 0.002 ^a^
Parietin	51.616 ± 0.490 ^b^	0.1036 ± 0.001 ^a^	0.1026 ± 0.001 ^a^	0.097 ± 0.001 ^a^	0.095 ± 0.001 ^a^	0.093 ± 0.001 ^a^	0.091 ± 0.001 ^a^
Ascorbic acid	4.451 ± 0.202 ^c^	1.541 ± 0.054 ^b^	0.853 ± 0.005 ^b^	0.406 ± 0.017 ^b^	0.215 ± 0.004 ^b^	0.110 ± 0.015 ^a^	0.105 ± 0.003 ^a^

Means with different letters in the same column indicate statistically significant differences among the extracts, parietin, and positive control (*p* < 0.05).

**Table 4:** Antibacterial activity (MIC and MBC values) of the acetone and methanol extracts of the lichen *P. deosaiense* as well as parietin.

Bacterial Species	Acetone ^aA^		Methanol ^aA^		Parietin ^aA^		Tetracycline ^B^	
	MIC	MBC	MIC	MBC	MIC	MBC	MIC	MBC
	mg/mL						µg/mL	
*Escherichia coli* ATCC 25922	>10	>10	>10	>10	>10	>10	4	6
*Proteus mirabilis* ATCC 12453	2.5	>10	1.25	>10	>10	>10	64	>128
*Pseudomonas aeruginosa* ATCC 10145	>10	>10	>10	>10	>10	>10	32	>128
*Staphylococcus aureus* ATCC 25923	>10	>10	>10	>10	>10	>10	2	3
*Staphylococcus aureus* MRSA ATCC 43300	>10	>10	>10	>10	>10	>10	<0.25	3
*Enterococcus faecalis* ATCC 29212	>10	>10	>10	>10	>10	>10	8	12
*Bacillus cereus* ATCC 11778	5	5	10	10	>10	>10	0.25	0.5

Different superscript letters mean statistically significant differences among the extracts, parietin, and positive control (*p* < 0.05).

**Table 5:** Percent (%) of the biofilm formation inhibition for tested extracts of the lichen *P. deosaiense* and parietin.

Bacterial Species	Acetone Extract ^a^					
	10 mg/mL	5 mg/mL	2.5 mg/mL	1.25 mg/mL	0.625 mg/mL	0.3125 mg/mL
*Proteus mirabilis* ATCC 12453	0	0	0	0	0	0
*Pseudomonas aeruginosa* ATCC 10145	92.1	0	0	0	0	0
*Staphylococcus aureus* ATCC 25923	92.0	91.1	87.9	90.6	66.8	54.2
	**Methanol Extract ^a^**					
*Proteus mirabilis* ATCC 12453	36.0	0	0	0	0	0
*Pseudomonas aeruginosa* ATCC 10145	81.7	4.7	0	0	0	0
*Staphylococcus aureus* ATCC 25923	90.3	89.7	85.2	73.4	68.9	63.7
	**Parietin ^a^**					
*Proteus mirabilis* ATCC 12453	92.5	25.6	0	0	0	0
*Pseudomonas aeruginosa* ATCC 10145	99.8	48.1	0	0	0	0
*Staphylococcus aureus* ATCC 25923	99.6	91.1	89.8	92.3	93.5	63.6

The same superscript letters mean no statistically significant differences among the extracts and parietin (*p* = 0.626).

**Table 6:** Percent (%) of the reduction in mature biofilm for tested extracts of the lichen *P. deosaiense* and parietin.

Bacterial Species	Acetone Extract ^a^					
	10 mg/mL	5 mg/mL	2.5 mg/mL	1.25 mg/mL	0.625 mg/mL	0.3125 mg/mL
*Proteus mirabilis* ATCC 12453	10.2	8.3	5.1	0	0	0
*Pseudomonas aeruginosa* ATCC 10145	28.8	23.7	20.2	0	0	0
*Staphylococcus aureus* ATCC 25923	69.8	63.6	59.8	62.0	52.0	0
	**Methanol extract ^a^**					
*Proteus mirabilis* ATCC 12453	20.4	11.9	0	0	0	0
*Pseudomonas aeruginosa* ATCC 10145	0	0	0	0	0	0
*Staphylococcus aureus* ATCC 25923	75.5	72.3	68.9	66.8	59.9	48.4
	**Parietin ^a^**					
*Proteus mirabilis* ATCC 12453	3.4	3.0	0	0	0	0
*Pseudomonas aeruginosa* ATCC 10145	59.58	0	0	0	0	0
*Staphylococcus aureus* ATCC 25923	85.3	73.7	56.4	51.85	47.3	39.4

The same superscript letters mean no statistically significant differences among the extracts and parietin (*p* = 0.993).

## Data Availability

The data presented in this study are available upon request from the corresponding author.
